# Finding Success at Work and at Home

**DOI:** 10.1200/GO.21.00261

**Published:** 2021-08-26

**Authors:** Shilpa Mukunda Chowdhry, Varun Kumar Chowdhry

**Affiliations:** Shilpa Mukunda Chowdhry, MD, Division of Geriatrics and Palliative Medicine, Department of Medicine, University at Buffalo, Buffalo, NY and Varun Kumar Chowdhry, MD, Department of Radiation Medicine, Roswell Park Comprehensive Cancer Center, Buffalo, NY

## TO THE EDITOR:

We read the report by Jiwnani et al^1^ on the impact of the COVID-19 pandemic on Indian physicians with great interest. We applaud the authors for highlighting challenges faced by physician families, particularly women, over the past year. These challenges have existed before the pandemic and will not end as we move past it. Although this study was conducted in India, we find that similar struggles are faced by physician mothers here in the United States. Drawing attention to these inequities will hopefully lead to advocacy on a policy level to address them.

Women's underestimation of household contributions and men's overestimation of efforts at home is not surprising. The same trend has been seen in the workplace. A meta-analysis evaluating perceptions of leadership skills in the workplace found that women underestimated their leadership skills, whereas men were more likely to overestimate their skills.^2^ Women endure the physical toll of having children and nourishing through breastfeeding, which is underappreciated both at work and at home. This became apparent to us as we welcomed our first child. Our practices in oncology are immensely data-driven, and we sought to apply this data-driven practice in caring for our son. Knowing the maternal and infant benefits to breastfeeding, we wanted to do our best to try to exclusively provide breastmilk for him. Despite a superficial encouragement of breastfeeding in medicine, new mothers are not provided a reduced clinical workload in most countries, and thus, time spent pumping at work could translate into longer working hours, 1 hour less with the baby, or the need to spend additional time working from home.

The authors note that unconventional solutions and support mechanisms are needed to help women in the workforce. We wanted to share some of our experiences as a dual-physician couple with children. We have found that balance of family life and work life requires an intricate game of tag team. One of us goes into work early and comes home early, whereas the other goes in later and stays later. One of us prepares dinner, while the other plays with our son. We switch roles when it comes time for the dishes. We support each other when we need to work from home, at the same time trying to draw lessons to teach our son about helping others through our work.

Another solution to our household duties draws from the economic principle of comparative advantage theory. The essence of this principle is that even if a country is capable of producing all the goods that it needs, factoring all the opportunity costs involved, it is still beneficial to seek trade from others. The same applies at home: we have found that we should not try to accomplish all household tasks, even if we are physically capable of completing them. We buy home-cooked meals prepared by members of our community, so we can finish our work and still have time to spend as a family. We routinely have our home cleaned. On weekends, we take help from grandparents to care for our son. These services became more challenging to continue during the pandemic, and we are fortunate that they have now become more available.

As a dual-physician couple, we face a daily balancing act of work and family obligations. We stay grounded in our busy lives through frequent reflection on the purpose behind our lives at work and at home. We were drawn to pursue careers in medicine not to simply prescribe medications, perform interventions, or amass a long list of publications: we want our work to improve the health and well-being of those we care for. As Clayton Christensen writes, “Don't worry about the level of individual prominence you have achieved; worry about the individuals you have helped become better people…Think about the metric by which your life will be judged, and make a resolution to live every day so that in the end, your life will be judged a success.”^3^ As physicians, we are privileged to guide our families, our patients, and all others around us to find happiness and health. This is the metric of success that we aspire to attain in everything that we do.

NOTE. A reply to this Correspondence was not provided.
